# FOXP2

**DOI:** 10.1002/wcs.1247

**Published:** 2013-08-13

**Authors:** Ron Nudel, Dianne F Newbury

**Affiliations:** Wellcome Trust Centre for Human Genetics, University of OxfordOxford, UK

## Abstract

The forkhead box P2 gene, designated *FOXP2*, is the first gene implicated in a speech and language disorder. Since its discovery, many studies have been carried out in an attempt to explain the mechanism by which it influences these characteristically human traits. This review presents the story of the discovery of the *FOXP2* gene, including early studies of the phenotypic implications of a disruption in the gene. We then discuss recent investigations into the molecular function of the *FOXP2* gene, including functional and gene expression studies. We conclude this review by presenting the fascinating results of recent studies of the *FOXP2* ortholog in other species that are capable of vocal communication. *WIREs Cogn Sci* 2013, 4:547–560. doi: 10.1002/wcs.1247

## INTRODUCTION

The story of the discovery of the *FOXP2* (forkhead box P2) gene (OMIM#605317) and the elucidation of its involvement in a speech and language disorder begins with the study of a multigenerational British family known as the KE family.

Studies of the KE family appeared in the scientific literature in the early 1990s. A first examination of the pedigree was carried out by Hurst et al.^1^ Various theories regarding the nature of the disorder have been proposed: Hurst et al.^1^ determined that affected family members suffered from developmental verbal dyspraxia, whereas Gopnik *et al*.^2^–^4^ proposed that the disorder involved an impairment of the grammatical competence of the affected family members, namely, in the morphological component thereof. The former view received further support from a thorough examination of 21 KE family members.^5^ The authors showed that the affected KE family members included in the study had, in addition to the morphological impairment, impaired processing, problems with articulation, and a severe orofacial dyspraxia^5^ (the reader is invited to refer to Table1 for definitions of various terms used throughout this paper). Moreover, affected KE family members, as a group, had lower verbal and performance IQ scores than unaffected KE family members.^5^ It was therefore evident that the affected KE family members had both a linguistic impairment and an extra-linguistic impairment.^5^ Given the inheritance pattern, it seemed likely that one gene was the cause of the disorder,^1^ but given the diverse nature of the impairment, the extent to which that gene was affecting language directly was unclear. Therefore, subsequent studies focused on defining the phenotypic nature of disorder, using both linguistic-behavioral and neuroimaging methods, on one hand, and on finding the gene itself, on the other hand.

**Table 1:** Definition of Various Terms Used in This Review

Term	Meaning
Allele	A given gene may have several variants across different individuals in a population; those variants are called the alleles of said gene.
Autosomal	Not linked to the X chromosome or the Y chromosome.
Centimorgan	A unit for measuring genetic linkage (how close regions on the same chromosome are).
Codon	A triplet of nucleotides (DNA bases) in the coding regions of a gene, which codes for an amino acid (or a stop signal during protein synthesis).
Co-segregation	The property of being inherited together within a pedigree.
Developmental verbal dyspraxia	A neurological disorder with an early onset affecting coordination of movements resulting in a linguistic impairment.
Dominant/recessive (inheritance pattern)	If one dysfunctional copy of a gene is enough to cause the disorder, where another functional copy is present, then the inheritance pattern of the particular disorder is said to be dominant. Otherwise the inheritance pattern of the disorder is said to be recessive.
fMRI	An MRI technique that measures neural activity in the brain based on the differences in the way oxygen-rich blood and oxygen-poor blood are affected by magnetic fields.
Gene family	A group of genes that share similarities in their sequences and functions.
Heterozygous/homozygous	A person carrying two identical alleles of a given gene on both chromosomes of the same type (one maternal and one paternal) is said to be homozygous for that gene. A person carrying two different alleles for the same gene is said to be heterozygous for that gene.
MicroRNA	A non-coding RNA molecule that regulates gene expression.
Missense mutation	A mutation which involves a change of a codon resulting in a codon which codes for a different amino acid.
MRI	An imaging technique that uses nuclear magnetic resonance (absorption and emission of electromagnetic radiation by nuclei affected by a magnetic field) to image the nuclei inside the subject's body.
Nonsense mutation	A mutation which involves a change of a codon resulting in a premature stop codon and, therefore, a truncated protein.
Orofacial dyspraxia	A neurological disorder in which voluntary nonverbal oral movements are impaired.
Parental imprinting effect	An effect by which the expression of a copy of a gene is dependent on which parent it was inherited from.
PET	An imaging technique which uses the radiation emitted by a radioactive tracer introduced into the living subject to produce a three-dimensional image.
Protein dimer	A protein complex formed by two protein subunits. If the units are identical, the dimer is called a homodimer; if the units are not identical, the dimer is called a heterodimer.
Transcription factor	A DNA-binding protein which regulates the expression of the genes it binds to.
Transition/transversion	A transition is a point mutation in the DNA of the form A↔G or C↔T. A transversion is a point mutation of the form C↔A,G or T↔A,G.

## DEFINING THE KE FAMILY PHENOTYPE

A 1998 study found that the affected KE family members were individually impaired on three tests: word repetition, nonword repetition, and simultaneous and sequential orofacial movements.^6^ The researchers further assessed the differences between some affected and some unaffected KE family members using two brain imaging techniques: positron emission tomography (PET) and magnetic resonance imaging (MRI). In the PET experiment, two affected KE family members and four normal control subjects were examined under two conditions: during scanning, the family members were asked to repeat words heard over earphones, compared to the baseline condition, in which they were asked to repeat a single specified word in response to hearing words that were reversed. It was found that some brain regions were underactive (compared to baseline levels) in the KE family members and some were overactive, compared to the normal controls. The underactive regions included the left supplementary motor area (SMA), the subjacent cingulate cortex on the left, and the left preSMA/cingulate cortex. These regions were activated compared to baseline levels in the controls but not in the two affected KE family members. The left sensorimotor face and mouth region was also less active in the affected KE family members than in the controls, but it was still active compared to baseline levels. The areas that were overactive in the two affected KE family members included the head and tail of the left caudate nucleus, the left premotor cortex with a ventral extension into Broca's area, and a left ventral prefrontal area.

Ten affected and seven unaffected KE family members were subsequently tested using MRI in order to try to find any structural differences in their brains that could account for the functional abnormalities in the affected members who were tested using PET. Several regions where affected members had significantly more or significantly less gray matter than unaffected members were identified: affected members had more gray matter in the lentiform nucleus and the angular gyrus (bilaterally in both cases), and less gray matter in preSMA/cingulate cortex, Broca's area and the caudate nucleus (bilaterally in the third case). The caudate nucleus was therefore implicated in both the PET and MRI analyses, and further examination showed that the affected family members had significantly smaller left and right caudate volumes than the unaffected family members.^6^ This finding was confirmed in another study,^7^ although it should be noted that the differences in the caudate nucleus volume were apparent at the group level (i.e., affected KE family members/unaffected KE family members/controls), and not at the individual level. Other brain regions identified in this study where affected KE family members differed in the amount of gray matter compared with unaffected members or controls included the putamen, the frontal operculum, the medial and lateral motor cortex, the cerebellum, and the planum temporal.^7^

A subsequent functional MRI (fMRI) study^8^ found that mainly two brain regions showed significant underactivation during silent verb generation, spoken verb generation, and word repetition, in affected KE family members: Broca's area and the putamen. The study also found that affected KE family members had fMRI activation of regions that are not usually involved in language-related tasks.

A behavioral analysis by Watkins et al.^9^ was carried out in an attempt to define the ‘core deficit’ in the affected KE family members. The cohort included 13 affected and 12 unaffected members of the KE family, and a group of 11 stroke patients with expressive aphasia. The study included intelligence tests and receptive and expressive language tests (including nonword repetition). Limb, oral, and facial musculature movements were assessed as well. The affected KE family members had significantly lower mean performance IQ than the unaffected family members and the aphasia patients. The affected family members were significantly impaired at receptive vocabulary compared with the two other groups, and together with the aphasia patients, they were significantly impaired at receptive grammar compared to the unaffected family members. The affected family members performed poorly compared with both other groups on a word repetition test, and compared with the unaffected family members on a nonword repetition test. The affected family members and the aphasia patients were significantly impaired at past tense production for both regular and irregular verbs compared with the unaffected family members. Similarly, the unaffected family members had significantly higher scores for orofacial praxis than the two other groups. While the affected family members and the aphasia patients performed similarly on some tests, namely, receptive grammar, nonword repetition, and past tense production, the aphasia patients had significantly higher performance IQ scores, as well as higher word repetition test scores. These and other differences discussed in the paper show the different aspects of a developmental versus an acquired speech and language disorder.^9^ The authors' analysis demonstrated that performance on a nonword repetition test was the best marker that discriminated the affected from the unaffected KE family members. Interestingly, nonword repetition has also been found to be a good phenotypic marker for specific language impairment.^10^ However, given the non-linguistic aspects of the KE family phenotype, the affected KE family members were not diagnosed with specific language impairment (see Box 1 for more information on specific language impairment).

**BOX 1 SPECIFIC LANGUAGE IMPAIRMENT**

Specific language impairment (SLI) is diagnosed when a child has major problems in the acquisition of language, despite showing normal development in all other areas.^11^ As reviewed by Stromswold,^12^,^13^ the results of several familial aggregation and twin studies support the hypothesis that SLI has a strong genetic component. In contrast to the speech and language disorder in the KE family, which is monogenic, SLI is thought to be a complex disorder, that is, a disorder which involves several genes and has a complex inheritance pattern.^14^ Regions on chromosomes 13,^15^ 16, and 19^16^,^17^ were identified in linkage studies of SLI. More recently, variants in three genes were found to be associated with SLI: *ATP2C2* (ATPase, Ca++ transporting, type 2C, member 2) and *CMIP* (c-Maf inducing protein),^18^ and *CNTNAP2* (contactin associated protein-like 2, OMIM#604569).^19^ The variants in *ATP2C2* and *CMIP* were significantly associated with nonword repetition ability in children affected by SLI, but not in the general population, which suggests that these genes are involved in phonological short-term memory in language-impaired children. Each of the two genes had an independent effect on nonword repetition ability. The variants in *CNTNAP2*, which were identified in another study, were also significantly associated with nonword repetition in children with SLI, as described in the text.

## A MOLECULAR ANALYSIS OF THE DISORDER AND THE DISCOVERY OF THE *FOXP2* GENE

While the behavioral and neuroimaging studies were taking place, researchers began looking for the gene that was the cause of the disorder in the KE family. The first analysis done by Hurst et al. suggested that the disorder from which half of the KE family members suffered was autosomal-dominant.^1^ A first genome-wide linkage analysis using 27 KE family members found strong evidence for linkage on the long arm of chromosome 7.^20^ Fine mapping of the region using additional markers implicated a 5.6 cM region in chromosomal band 7q31.^20^ This region has been designated SPCH1 (OMIM#602081). A subsequent study of the SPCH1 region^21^ used newly generated polymorphic markers, which allowed for a more accurate linkage analysis.^21^ In addition to that, two subjects suffering from language problems were also included in this study. One of those subjects, CS, also had verbal dyspraxia, thus making his phenotype very similar to that of the affected KE family members. CS had a chromosomal translocation with a breakpoint that was mapped to a single clone inside SPCH1,^21^ thus providing further evidence for the involvement of this region in speech and language disorders. The refinement of the exact breakpoint showed that it fell inside a gene which was designated *FOXP2*, due to structural similarities shared with other FOX proteins (transcription factors that have a characteristic forkhead DNA-binding domain).^22^ Furthermore, an inspection of the coding regions of the *FOXP2* gene in the KE family resulted in the detection of a G to A transition in exon 14 which co-segregated perfectly with the disorder.^22^ This is a missense mutation that results in an arginine-to-histidine substitution in the forkhead DNA-binding domain of FOXP2,^22^ at position 553 of the protein (R553H). The authors explain that this arginine residue is important for the function of the forkhead domain of the protein, and, consequently, the mutation in the KE family disrupts the DNA-binding properties of this domain, thus rendering FOXP2 dysfunctional.^22^ All affected KE family members were heterozygous for this mutation, which supports the original finding that the disorder is dominant. Given their molecular findings, the authors propose that *FOXP2* haplo-insufficiency in the brain at a key stage of embryogenesis leads to abnormal development of neural structures that are important for speech and language.^22^ The KE mutation was found to be influencing the intracellular localization of the protein, its DNA-binding capacity, and its transactivation capacity in a human neuron-like cell line that had been transfected with the R553H construct.^23^ One study that looked into this found that when cells were transfected only with the mutant construct, the protein showed abnormal intracellular localization: significant amounts of it were found in the cytoplasm, while the wild-type protein is found predominantly in the nucleus^23^; however, in cells that had both the mutant and wild-type FOXP2 protein (which is representative of the situation in the KE family), a heterodimer of the mutant/wild-type FOXP2 was found in the nucleus. Thus it is possible that the disorder observed in the KE family might be caused by either the mutant protein's abnormal localization in the cytoplasm and/or the formation of heterodimers in the nucleus.^24^

A mutation screening of children with verbal dyspraxia found three previously unreported exonic variants in the *FOXP2* gene.^25^ Two of the three variants did not co-segregate with affection status in the probands' respective families. However, one of these variants, a nonsense mutation yielding a stop codon at position 328 of the protein (R328X), did co-segregate with the disorder in the proband's family: an affected sibling and the mother, who had a history of speech problems, also had the mutation, whereas the father, who had normal speech, did not have it. This mutation leads to a dramatic truncation of the FOXP2 protein, thereby resulting in a protein with a lack of specific functional domains, including the forkhead DNA-binding domain.^25^ A functional study of this mutation revealed that the protein was predominantly found in the cytoplasm (instead of the nucleus, as expected for FOXP2) and showed no DNA-binding or transactivation capacities.^23^ As in the case of the affected KE family, the proband and affected family members in this study were heterozygous for the nonsense mutation.

A deletion in chromosome 7q31-q32 which included *FOXP2* was reported for a child with oromotor and verbal dyspraxia and developmental delay.^26^ Possible imprinting effects for *FOXP2* have also been reported in the context of developmental verbal dyspraxia, in which the absence of paternally inherited *FOXP2* was associated with the condition.^27^ However, another study showed that a loss of maternally inherited *FOXP2* still resulted in verbal dyspraxia,^28^ and it is now believed that *FOXP2* is not imprinted.^29^ It should be pointed out that while developmental verbal dyspraxia is often present in children with a dysfunctional copy of the *FOXP2* gene, not all cases of developmental verbal dyspraxia are the result of such disruptions in *FOXP2*. Table2 summarizes the cases discussed above.

**Table 2:** List of Case with *FOXP2* Disruptions Discussed in This Review

Case Description	Reference to the Paper(s) Describing the Genetic Cause for the Disorder
The affected members of the KE family, who had a diverse phenotype caused by a point mutation in *FOXP2*.	Lai et al.^22^
CS, who had verbal dyspraxia resulting from a translocation disrupting the *FOXP2* gene.	Lai et al.^22^
Three non-synonymous mutations in children with developmental verbal dyspraxia.	MacDermot et al.^25^
Oromotor and verbal dyspraxia and developmental delay in a child carrying a *FOXP2* deletion.	Zeesman et al.^26^
A loss of paternal *FOXP2* in children with developmental verbal dyspraxia.	Feuk et al.^27^
A loss of maternal *FOXP2* resulting in verbal dyspraxia.	Rice et al.^28^

Several studies have investigated the possibility that normal genetic variation, as opposed to genetic mutation, across *FOXP2* may be involved in the etiology of neurological disorders. An association analysis and mutation screen of the *FOXP2* gene was done on families with autism or specific language impairment, but no evidence for the direct involvement of *FOXP2* in either of the disorders was found.^30^ However, some genetic variants in *FOXP2* showed association with some language and reading abilities and sequential motor activities in a dyslexia cohort.^31^ In addition to the studies involving subjects with impaired speech and/or language abilities, a study of genetic variants inside the *FOXP2* gene in healthy subjects found some associations with variations in activation during reading (detected with fMRI) in the left inferior frontal gyrus and the left precentral gyrus.^32^

Interestingly, a genetic variant in *FOXP2* was found to be associated with poverty of speech in schizophrenia patients,^33^ and another genetic variant was found to be associated with reduced gray matter concentrations in schizophrenia patients, in brain regions related to the disease.^34^ See Ref 35 for a review that includes further studies of the phenotypic implications of *FOXP2* and *FOXP1* (discussed below) disruptions.

## THE FUNCTION OF THE *FOXP2* GENE

### *FOXP2* Expression

As has been shown, disruptions in the *FOXP2* gene cause both linguistic and non-linguistic deficits. What mechanism might explain this fact? The FOXP2 protein is a transcription factor.^22^,^36^ Transcription factors are proteins that bind to the genomic DNA and can either activate or repress specific genes, i.e., they can increase or decrease the production of transcripts of a gene. Strong *FOXP2* expression was detected in fetal brains.^22^ Murine *Foxp2* expression was detected in the lungs and in neural, intestinal and cardiovascular tissues during development.^36^ *FOXP2* expression was also detected in the caudate nucleus of adult human brains,^37^ a brain region implicated in the neuroimaging studies of the KE family. In the developing human brain, *FOXP2* expression can be detected from as early as the 44th day of gestation.^38^ *FOXP2* expression is first detected in the midline of the hindbrain, and subsequently becomes more complex as the embryonic development progressed.^38^ *FOXP2* expression was detected only in some brain regions, including the cerebellum, thalamus, caudate nucleus, and the putamen, and was sometimes limited to specific structures in those regions, e.g., Purkinje cells in the cerebellum.^38^ Some of these brain regions, such as the caudate nucleus, the putamen, and the cerebellum, were previously implicated as regions in which affected KE family members differed from unaffected KE family members or controls in terms of structural abnormalities^7^ (see above).

### *FOXP2* Targets

It is thus clear that, as a transcription factor expressed in the brain, *FOXP2* can potentially affect a variety of genes that could be relevant to speech, language, and/or motor control. In theory, it could also be influencing each of these phenotypes independently. Researchers thus set out to find the genes that FOXP2 regulates, i.e., its targets. One study^39^ looked for FOXP2 targets in cells derived from the basal ganglia and the inferior frontal cortex of human fetal brains (during midgestation), as well as cells derived from human lung tissue, as *FOXP2* shows high expression in the lung.^36^ This study identified 175 FOXP2 targets in the basal ganglia and 144 targets in the inferior frontal cortex, with a 24% overlap of inferior frontal cortex genes over basal ganglia genes. Additionally, 141 genes and 110 other target genes were specific to the basal ganglia and the inferior frontal cortex, respectively. These may represent targets of region-specific FOXP2 regulation.^39^ 192 targets were identified in lung tissue, with a 47% and 37% overlap between targets in the lung and targets in the basal ganglia and inferior frontal cortex, respectively.

Another study looked for FOXP2 targets in human neuron-like cells.^40^ This study identified 303 FOXP2 targets. Results of gene ontology analyses of the targets identified in the basal ganglia and the inferior frontal cortex^39^ and of the 100 most significant targets in neuron-like cells^40^ suggested that FOXP2 targets have various roles in the development of the nervous system and signal transduction, including synaptic transmission.^39^,^40^ FOXP2 was shown to be acting as a repressor for the majority of the targets for which expression had been measured.^39^,^40^ The overlap between the results reported in Spiteri et al.^39^ and Vernes et al.^40^ was remarkable: 29% of the targets identified in the basal ganglia and 30% of the targets identified in the inferior frontal cortex as reported in the former paper represented 14–19% of the potential targets identified in the latter paper.^40^

### The Role of *FOXP2* in Neural Development

Studies into the specific involvement of *FOXP2* in neural development have also been carried out. One investigation showed that the knockdown of Foxp2 expression in the developing mouse cortex prevented the transition of radial precursors suggesting that murine *Foxp2* regulates neurite (axon or dendrite) outgrowth during brain development.^41^ Similarly, another study found that ectopic expression of *Foxp2* in the embryonic mouse neocortex delays neurite outgrowth and impairs radial migration, a process which is normally repressed through microRNA mediation.^42^ Interestingly, while both human *FOXP2* and mouse *Fopx2* have been shown to regulate the genesis of intermediate progenitors and neurons from multipotent radial precursors, only the human *FOXP2*, when overexpressed, enhances the genesis of intermediate progenitors and neurons.^43^ In this context, the FOXP2 protein with the amino acid change found in affected KE family members was shown to cause increased radial precursors and decreased intermediate progenitors and neurons.^43^ Taken together, these findings support those of FOXP2 targets and provide further support to the hypothesis that *FOXP2* is involved in neural development, and, particularly, in neurite outgrowth.

#### CNTNAP2

A good example of a gene in a pathway through which *FOXP2* may be influencing language is that of *CNTNAP2* (contactin associated protein-like 2, OMIM#604569). *CNTNAP2* encodes a transmembrane protein, CASPR2, which is found at the nodes of Ranvier (gaps in the myelin sheaths insulating the axons of neurons).^44^ Additionally, CASPR2 appears to be involved in cortical development and neuroblast migration and laminar organization in humans.^45^ *CNTNAP2* shows enriched expression in brain regions that are important for language, and its expression pattern in the human brain is remarkably different from its expression pattern in the rodent brain.^46^

Mutations in *CNTNAP2* have been implicated in epilepsy, language regression and mental retardation,^45^ and Tourette's syndrome,^47^ and variants in the gene have been shown to be associated with age at first word in probands with autism.^48^

A study by Vernes et al.^19^ found that *CNTNAP2* was a FOXP2 target, and that it was down-regulated by it. Moreover, this study also found an association between variants in *CNTNAP2* and nonword repetition in probands with specific language impairment: nine intronic SNPs located between exons 13 and 15 of *CNTNAP2* were significantly associated with nonword repetition.^19^ An association for one of those SNPs with nonword repetition was replicated in a dyslexia cohort.^31^ Interestingly, a recent association study of early language proficiency in the general population found that the variants in *CNTNAP2* implicated in studies of specific language impairment^19^ and autism^48^ were also associated with the early stages of language development in children from the general population.^49^

More recently, an exome-sequencing study of sporadic autism spectrum disorders identified a potentially deleterious missense mutation in *CNTNAP2* in a proband who also had a *de novo* mutation in *FOXP1* (forkhead box P1).^50^ This is interesting as both of these genes relate to *FOXP2*: *CNTNAP2* is regulated by *FOXP2*, and *FOXP1* interacts with it. Findings, such as this, assist in the further characterization of pathways involving FOXP2 and assist in the identification of major players thereof. See Box 2 for more information on the possible interaction between *FOXP2* and *FOXP1*.

Thus, *CNTNAP2*, a FOXP2 target, appears to be involved in several neurodevelopmental disorders in which language is impaired. This example shows how *FOXP2* can be influencing various overlapping pathways in various disorders. Considering the fact that FOXP2 has many neural targets,^39^,^40^ it is not surprising that a disruption in *FOXP2* can manifest itself as a very complex phenotype.

## *FOXP2* IN OTHER SPECIES

*FOXP2* is not found only in humans; *FOXP2* orthologs are found in other vertebrates, and the FOXP2 protein is conserved across different species.^56^ If one disregards two polyglutamine stretches, which vary in different species, the *FOXP2* protein is highly conserved across different species.^57^ It is thus possible to study the function of the gene in other species, many of which, like humans, communicate vocally.

**BOX 2 A POSSIBLE INTERACTION BETWEEN *FOXP2* AND *FOXP1***

*FOXP2* and *FOXP1* (OMIM#605515) both belong to the same gene family, the forkhead/winged-helix family of transcription factors, and their proteins share a high degree of similarity in their DNA-binding domain.^36^ Like *FOXP2*, *FOXP1* is also expressed in neural tissue during development.^36^ Both proteins have two highly homologous repression domains.^51^ An expression study of *FOXP2* and *FOXP1* in developing and adult mice brains and in human fetal brain tissue found that while both genes were expressed in the brain, they each had their own distinct expression pattern with regards to different brain regions, with the exception of the basal ganglia.^52^ Both genes were expressed in the cortex, albeit in different layers.^52^ Interestingly, a study of *FOXP2* and *FOXP1* expression in human and zebra finch (a songbird) brains found that both genes were expressed in brain circuits related to song learning and production.^53^ Moreover, *FOXP1*, in contrast to that of *FOXP2*, consistently showed a difference in expression levels between males and females in sexually dimorphic brain regions, in that males had high levels of *FOXP1* expression in the song-related brain regions, whereas the corresponding regions in the female brain did not.^53^ Expression patterns of both genes in human and zebra finch brain appeared to be highly similar.^53^ The structural and functional similarities between the two genes and the partial overlap in expression patterns in brain regions important for language may suggest a possible interaction between them. More recently, disruptions in *FOXP1* have been implicated in speech delay, intellectual disability, and autism,^50^,^54^,^55^ and more cases are reviewed in Ref 35.

## Sequence Conservation in Other Species

The human and mouse *FOXP2* orthologs differ at only three amino acids, whereas the human and chimpanzee orthologs differ at two of those three amino acids.^57^ These two amino acid substitutions [threonine-to-asparagine at position 303 (T303N) and asparagine-to-serine at position 325 (N325S)] are both found in exon 7 of *FOXP2*. The asparagine-to-serine substitution occurred independently in some carnivore and bat species,^56^,^58^ which suggests that this substitution alone cannot account for any human-specific functions of *FOXP2*. In addition to that, these two amino acid substitutions were not found in whales, which are also vocal-learning mammals.^56^ That said, these two amino acid substitutions were not found to be polymorphic in humans.^56^,^57^ These substitutions may have functional consequences, and they are likely to have been positively selected in humans.^56^,^57^

As *FOXP2* is involved in speech and language in humans, and it is found in other species, the question was raised of whether any changes in *FOXP2* could be found in animal species with more complex forms of vocal communication (i.e., ones in which learning is involved) compared with species with simpler forms of vocal communication. Webb et al.^59^ compared the sequence of exon 7 in the *FOXP2* gene of song-learning birds with that of non-song-learning birds, and examined whether other vocal-learning mammals such as whales and dolphins shared any of the amino acid substitutions that were thought to be human-specific. No difference in the amino acid sequence encoded by exon 7 of *FoxP2* between song-learning birds and non-song-learning birds was found,^59^ and the sequence did not differ from that of the mouse. Similarly to the case of whales, the dolphin amino acid sequence of exon 7 did not contain the two amino acid substitutions previously found in humans.^59^ However, it was found that whales and dolphins shared three amino acid substitutions, in contrast to the hippopotamus, their closest relative, which had an amino acid sequence identical to that of the mouse, and the human threonine-to-asparagine substitution was flanked by two substitutions in the whale and dolphin sequences.^59^ Interestingly, *FOXP2* orthologs in echolocating bats were quite diverse in their sequences, and some variation in exons 7 and 17 of the bat *FoxP2* seemed to be correlated with echolocation type and phylogenetic boundaries, suggesting that *FoxP2* may have played a role in the development of these echolocation systems.^58^

A 2007 study by Krause et al.^60^ found the two amino acid substitutions, T303N and N325S, in two Neanderthal samples. The authors argue that, given their data, it is likely that changes in *FOXP2* and the selective sweep originated in the common ancestor of Neanderthals and modern humans, suggesting that they took place sometime between 300,000 and 400,000 years ago.^60^ However, this view was challenged by Coop et al.,^61^ who also argued that further experiments were needed in order to rule out human contamination of the Neanderthal samples. An alternative suggested by Ptalk et al. is that two selective sweeps possibly occurred: one before the human–Neanderthal split (during which the two amino acid substitutions were fixed) and one after the split occurring only in humans and unrelated to the two amino acid substitutions.^62^ The authors show that this scenario is consistent with the linkage disequilibrium and Neanderthal data. The consequences thereof may mean that some unknown human-specific variants have been positively selected nonetheless.^62^ One candidate for this potential selective sweep is a variant in a regulatory sequence in *FOXP2* that is not present in Neanderthals.^63^ A study that compared the regulatory function of human *FOXP2* and chimpanzee *FoxP2* in human neuronal cells found 116 genes that were significantly differentially regulated by *FOXP2* and *FoxP2*.^64^ The authors suggest that some of these human-specific differential targets may be important in higher cognitive functions.^64^

## Conservation of *FOXP2* Expression Patterns in Other Species

Several studies investigated the expression patterns of *FOXP2* in neural tissue in a variety of species. Lai et al.^38^ compared the expression patterns of *FOXP2* in the developing human and mouse brains. Their results indicated high conservation of expression patterns at onset in the brains of both species, with regards to both the timing and tissue distribution. Even as brain development progressed, both expression patterns continued to resemble each other (it should be noted that the studying of human embryonic expression was confined to early gestation). Teramitsu et al. found highly similar expression patterns in human and zebra finch brains (discussed in more detail in Box 2).^53^

A study of *FoxP2* expression in avian vocal learners and non-learners found that striatal expression was similar between birds and mammals, whereas pallial expression was not.^65^ Another interesting finding from this study is that *FoxP2* expression levels in Area X (a brain region involved in the acquisition and learning of songs in song-birds which is part of the basal ganglia loop) increased in young zebra finches and in adult canaries when the former learned to imitate song and when the latter remodeled their songs.^65^

A study of *FoxP2* expression in zebrafish found that the expression pattern in the brain of the zebrafish was similar to that of humans and included regions that correspond to brain regions which showed abnormalities in the affected KE family members.^66^

Other studies have investigated *FoxP2* expression across various species. These include a study of the rat brain, which found high levels of expression in the striatum of the basal ganglia during brain development,^67^ a study of the monkey brain, which found a mosaic FoxP2 expression pattern in the caudate nucleus and putamen (which decreased during development, starting with the putamen) and the developing cerebral cortex,^68^ and a study of *Foxp2* expression in four species of mice, which also found a high and heterogeneous *Foxp2* expression pattern in the caudate nucleus and putamen across species.^69^

The high degree of conservation in the expression patterns in neural tissue and amino acid sequence of *FOXP2* orthologs across various species suggest that *FOXP2* may have a more general role in the development of the nervous system.

## Functional Studies of *FOXP2* in Other Species

*FOXP2* has been the subject of several functional studies using animal models. Shu et al.^70^ disrupted one or two copies of the *Foxp2* gene in mice, thus producing heterozygous mice or knockout mice, respectively. The knockout mice showed severe motor impairment, and died prematurely.^70^ Both the heterozygous and the knockout mice had a significantly reduced number of ultrasonic vocalizations (calls that are elicited by the pups when removed from their mothers) compared with the wild-type mice, despite the fact that apparatus for the production of these vocalizations was found to be normal in both heterozygous and knockout mice.^70^ Cerebral deficits were also found in all mice carrying the *Foxp2* disruption.^70^Fujita et al.^71^ studied mice that had been genetically engineered to carry a mutation in *Foxp2* corresponding to the mutation found in the affected KE family members. Mice homozygous for this mutation showed severe motor abnormalities.^71^ Heterozygous mice showed modest impairment in ultrasonic vocalization, whereas homozygous mice showed severe impairment.^71^ The *Foxp2* mutation also prevented the normal development of the cerebellum in homozygous mice and the normal maturation of dendrites in the Purkinje neurons in both homozygous and heterozygous mice.^71^Groszer et al. also studied mice carrying this mutation (using an independent mouse generated for this study), and found that it caused cerebellar abnormalities in homozygous mice and deficits in motor-skill learning, rather than gross motor skills as previously reported, and abnormal striatal synaptic plasticity in heterozygous mice, including a loss of long-term synaptic depression.^72^ In terms of their vocalizations, homozygous mice produced significantly fewer distress calls than the heterozygous and wild-type mice, and significantly more clicks than the wild-type mice.^72^ However, a study of very young mouse pups carrying either of two mutations (one corresponding to the missense mutation found in affected KE family members,^22^ and another corresponding to the nonsense mutation found in a proband and his mother^25^) showed that mouse pups carrying these mutations did not significantly differ from wild-type mice in terms of their vocalizations, when the pups were subject to isolation or distress.^73^ Since the mice pups were only 4 days old, an age at which they are still deaf, the conclusions of this study pertain to the innate production mechanisms of the vocalizations, and not potentially learned behavior, in contrast to previous studies.^73^

A recent study^74^ examined auditory-motor association learning in heterozygous mice carrying the same two types of mutation described above. The mice had to learn to associate a certain sound with a motor activity required to cross a hurdle. Mice carrying the missense mutation were able to learn at a slower rate than wild-type mice, but ultimately reached the same performance level as that of the wild-type mice, whereas mice carrying the nonsense mutation also learned slowly, never reaching the same performance level as that of the wild-type mice.^74^ In another study, mice carrying the KE family mutation were shown to have abnormal striatal activity in terms of the firing rate of the neurons, as well as changes in striatal plasticity during motor-skill learning, compared to wild-type mice.^75^

Haesler et al.^76^ specifically reduced FoxP2 levels in the Area X region in the brains of zebra finches using RNA interference. In their previous study, Haesler et al. discovered that *FoxP2* expression levels in Area X increased during song learning.^65^ In their subsequent study, in which FoxP2 levels were purposely reduced in Area X in male zebra finches, Haesler et al. found that the zebra finches that had their FoxP2 levels reduced (knockdown zebra finches) exhibited poor song-imitation skills (young males learn their songs from adult male tutors), which typically included syllable omissions, imprecise copying of syllable duration, and inaccurate imitation of spectral characteristics.^76^ Knockdown zebra finches also displayed more variability in syllable production (i.e., when producing the same syllable on several occasions).^76^ Another study examined *FoxP2* regulation in Area X of adult zebra finches, in a social context, during directed and undirected singing, i.e., when males sing during social interactions and when they are alone or do not sing toward a conspecific, respectively.^77^ It should be noted that this study focused on *FoxP2* regulation in Area X during adult song production and not song learning. Interestingly, *FoxP2* in Area X was down-regulated in the context of undirected singing, compared to that of directed singing.^77^ These results, which show that *FoxP2* is down-regulated during undirected singing in adult zebra finches in a brain region usually associated with song learning in young zebra finches, may suggest a role for *FoxP2* beyond the developmental stages of vocal control circuits in the brain.^77^ At the protein level, FoxP2 levels in Area X have been shown to decrease after both directed and undirected singing. However, a negative (and nonsignificant) correlation between FoxP2 levels and amount of singing was found only in the context of undirected singing.^78^

A subsequent study examined *FoxP2* regulation in Area X in both hearing and deafened zebra finches.^79^ Deafening resulted in disrupted development but did not affect basal FoxP2 levels.^79^ Interestingly, *FoxP2* was down-regulated in both hearing and deafened young zebra finches, similar to the case of adult zebra finches, after they were allowed to sing for 2 h, suggesting that the *FoxP2* regulation did not solely depend on auditory feedback, although only in the hearing birds did the down-regulation depend on the amount of singing, suggesting a more complex regulatory mechanism.^79^

### Animal Studies of the Humanized *FOXP2*

In a recent study a humanized version of the *FOXP2* gene was studied in mice. The two previously discussed amino acid substitutions that are found in humans (but not in chimpanzees) (T303N and N325S) were introduced into the endogenous murine *Foxp2*.^80^ Mice carrying two humanized alleles were compared to mice carrying one disrupted humanized allele (either R553H or R328X) and one wild-type murine allele. The former exhibited reduced exploratory behavior compared with the latter, but no other significant different was found in almost 300 measurements of assessing a variety of physiological systems, suggesting that the humanized allele influences mainly the brain.^80^

Further investigations of the effect the humanized *FOXP2* might have on the mouse brain found the following: a reduction in dopamine levels in humanized *FOXP2* mice compared to the wild-type and heterozygous mice; longer dendritic trees of medium spiny neurons in the striatum of humanized *FOXP2* mice compared with the wild-type and heterozygous mice; long-term synaptic depression was higher in medium spiny neurons of humanized *FOXP2* mice than in those of wild-type mice; altered frequencies of ultrasonic vocalizations in humanized *FOXP2* mice compared with wild-type mice.^80^ As the humanized *FOXP2* was not found to be influencing physiological measurements unless they were related to the central nervous system, it is likely that the two amino acid substitutions in question affect the brain circuits in the mouse, of which the corresponding regions in the human brain could arguably be relevant to speech and language.^80^ An additional finding in this study was that 34 genes showed differential expression between humanized *FOXP2* mice and wild-type mice.^80^

A subsequent study examined whether the changes in long-term synaptic depression and dendritic length occur in other brain regions, and concluded that the humanized *FOXP2* increased dendritic length only in neurons that form part of the cortico-basal ganglia circuits, and that it influences synaptic plasticity in medium spiny neurons, but not in Purkinje neurons.^81^

## CONCLUSION

The *FOXP2* gene is the first gene found to be involved in a speech and language disorder. The disorder was first observed and studied in a British family known as the KE family, but similar, independent cases have since been reported. Various disruptions in the *FOXP2* gene have been reported, and the conditions caused by said disruptions tend to include a broad spectrum of deficits, including: speech and language problems, verbal dyspraxia, low performance IQ, developmental delay, and brain abnormalities. The FOXP2 protein acts as a transcription factor, and has been shown, for the main part, to repress the expression of neural targets. A thoroughly studied example of a gene repressed by FOXP2, *CNTNAP2*, has been implicated in disorders such as specific language impairment and autism, among others, which sheds some light on the mechanism through which *FOXP2* may be influencing speech and language. FOXP2 is a highly conserved protein and exists in many other vertebrates. Studies of *FOXP2* function in species that communicate vocally and/or are vocal learners show that there exists a high degree of conservation in expression patterns across different species, and that FOXP2 is involved in brain circuits that are important for vocal learning and communication. The human *FOXP2* ortholog contains amino acid substitutions which, when introduced into the mouse, have been shown to affect the function of the gene in the brain. Given the evidence from the wide variety of studies that have been performed at various levels, from the gene and up to the brain, it is clear why a disruption in *FOXP2* may have a cascade of different effects: it can cause changes in the regulation of FOXP2 target genes, many of which are expressed in the brain. Functional studies of *FOXP2* have shown that the gene plays a crucial role in the development of several brain regions that are known to be part of speech and language-related circuits. Molecular studies of the effects of *FOXP2* disruptions in individuals with a speech and language disorder show that its function is considerably affected, which may result in the abnormal development of important brain regions. The exact mechanisms and pathways of FOXP2 and its neuronal targets and the way in which they affect speech and language are not yet completely clear, but continuing work is being carried out by scientists from various disciplines, from genetics to neuroscience, in order to shed some light thereon. Future studies may focus on the genes that regulate *FOXP2* as well as continue to study the genes that *FOXP2* regulates. The genetic and biochemical study of these complex genetic pathways, as well as functional studies in vocal learning species will allow us to better understand the biological foundations of human language.

## References

[1] Hurst JA, Baraitser M, Auger E, Graham F, Norell S (1990). An extended family with a dominantly inherited speech disorder. Dev Med Child Neurol.

[2] Gopnik M (1990). Feature-blind grammar and dysphasia. Nature.

[3] Gopnik M (1990). Genetic basis of grammar defect. Nature.

[4] Gopnik M, Crago MB (1991). Familial aggregation of a developmental language disorder. Cognition.

[5] Vargha-Khadem F, Watkins K, Alcock K, Fletcher P, Passingham R (1995). Praxic and nonverbal cognitive deficits in a large family with a genetically transmitted speech and language disorder. Proc Natl Acad Sci U S A.

[6] Vargha-Khadem F, Watkins KE, Price CJ, Ashburner J, Alcock KJ, Connelly A, Frackowiak RS, Friston KJ, Pembrey ME, Mishkin M (1998). Neural basis of an inherited speech and language disorder. Proc Natl Acad Sci U S A.

[7] Watkins KE, Vargha-Khadem F, Ashburner J, Passingham RE, Connelly A, Friston KJ, Frackowiak RS, Mishkin M, Gadian DG (2002). MRI analysis of an inherited speech and language disorder: structural brain abnormalities. Brain.

[8] Liegeois F, Baldeweg T, Connelly A, Gadian DG, Mishkin M, Vargha-Khadem F (2003). Language fMRI abnormalities associated with FOXP2 gene mutation. Nat Neurosci.

[9] Watkins KE, Dronkers NF, Vargha-Khadem F (2002). Behavioural analysis of an inherited speech and language disorder: comparison with acquired aphasia. Brain.

[10] Bishop DV, North T, Donlan C (1996). Nonword repetition as a behavioural marker for inherited language impairment: evidence from a twin study. J Child Psychol Psychiatry.

[11] Bishop DV (2006). What Causes Specific Language Impairment in Children?. Curr Dir Psychol Sci.

[12] Stromswold K (1998). Genetics of spoken language disorders. Hum Biol.

[13] Stromswold K (2001). The heritability of language: a review and metaanalysis of twin, adoption, and linkage studies. Language.

[14] Newbury DF, Bishop DV, Monaco AP (2005). Genetic influences on language impairment and phonological short-term memory. Trends Cogn Sci.

[15] Bartlett CW, Flax JF, Logue MW, Vieland VJ, Bassett AS, Tallal P, Brzustowicz LM (2002). A major susceptibility locus for specific language impairment is located on 13q21. Am J Hum Genet.

[16] The SLI consortium (2002). A genomewide scan identifies two novel loci involved in specific language impairment. Am J Hum Genet.

[17] The SLI consortium (2004). Highly significant linkage to the SLI1 locus in an expanded sample of individuals affected by specific language impairment. Am J Hum Genet.

[18] Newbury DF, Winchester L, Addis L, Paracchini S, Buckingham LL, Clark A, Cohen W, Cowie H, Dworzynski K, Everitt A (2009). CMIP and ATP2C2 modulate phonological short-term memory in language impairment. Am J Hum Genet.

[19] Vernes SC, Newbury DF, Abrahams BS, Winchester L, Nicod J, Groszer M, Alarcon M, Oliver PL, Davies KE, Geschwind DH (2008). A functional genetic link between distinct developmental language disorders. N Engl J Med.

[20] Fisher SE, Vargha-Khadem F, Watkins KE, Monaco AP, Pembrey ME (1998). Localisation of a gene implicated in a severe speech and language disorder. Nat Genet.

[21] Lai CS, Fisher SE, Hurst JA, Levy ER, Hodgson S, Fox M, Jeremiah S, Povey S, Jamison DC, Green ED (2000). The SPCH1 region on human 7q31: genomic characterization of the critical interval and localization of translocations associated with speech and language disorder. Am J Hum Genet.

[22] Lai CS, Fisher SE, Hurst JA, Vargha-Khadem F, Monaco AP (2001). A forkhead-domain gene is mutated in a severe speech and language disorder. Nature.

[23] Vernes SC, Nicod J, Elahi FM, Coventry JA, Kenny N, Coupe AM, Bird LE, Davies KE, Fisher SE (2006). Functional genetic analysis of mutations implicated in a human speech and language disorder. Hum Mol Genet.

[24] Mizutani A, Matsuzaki A, Momoi MY, Fujita E, Tanabe Y, Momoi T (2007). Intracellular distribution of a speech/language disorder associated FOXP2 mutant. Biochem Biophys Res Commun.

[25] MacDermot KD, Bonora E, Sykes N, Coupe AM, Lai CS, Vernes SC, Vargha-Khadem F, McKenzie F, Smith RL, Monaco AP (2005). Identification of FOXP2 truncation as a novel cause of developmental speech and language deficits. Am J Hum Genet.

[26] Zeesman S, Nowaczyk MJ, Teshima I, Roberts W, Cardy JO, Brian J, Senman L, Feuk L, Osborne LR, Scherer SW (2006). Speech and language impairment and oromotor dyspraxia due to deletion of 7q31 that involves FOXP2. Am J Hum Genet Part A.

[27] Feuk L, Kalervo A, Lipsanen-Nyman M, Skaug J, Nakabayashi K, Finucane B, Hartung D, Innes M, Kerem B, Nowaczyk MJ (2006). Absence of a paternally inherited FOXP2 gene in developmental verbal dyspraxia. Am J Hum Genet.

[28] Rice GM, Raca G, Jakielski KJ, Laffin JJ, Iyama-Kurtycz CM, Hartley SL, Sprague RE, Heintzelman AT, Shriberg LD (2012). Phenotype of FOXP2 haploinsufficiency in a mother and son. Am J Hum Genet: Part A.

[29] Thomas AC, Frost JM, Ishida M, Vargha-Khadem F, Moore GE, Stanier P (2012). The speech gene FOXP2 is not imprinted. J Med Genet.

[30] Newbury DF, Bonora E, Lamb JA, Fisher SE, Lai CS, Baird G, Jannoun L, Slonims V, Stott CM, Merricks MJ (2002). FOXP2 is not a major susceptibility gene for autism or specific language impairment. Am J Med Genet.

[31] Peter B, Raskind WH, Matsushita M, Lisowski M, Vu T, Berninger VW, Wijsman EM, Brkanac Z (2011). Replication of CNTNAP2 association with nonword repetition and support for FOXP2 association with timed reading and motor activities in a dyslexia family sample. J Neurodev Disord.

[32] Pinel P, Fauchereau F, Moreno A, Barbot A, Lathrop M, Zelenika D, Le Bihan D, Poline JB, Bourgeron T, Dehaene S (2012). Genetic variants of FOXP2 and KIAA0319/TTRAP/THEM2 locus are associated with altered brain activation in distinct language-related regions. J Neurosci.

[33] Tolosa A, Sanjuan J, Dagnall AM, Molto MD, Herrero N, de Frutos R (2010). FOXP2 gene and language impairment in schizophrenia: association and epigenetic studies. BMC Med Genet.

[34] Spaniel F, Horacek J, Tintera J, Ibrahim I, Novak T, Cermak J, Klirova M, Hoschl C (2011). Genetic variation in FOXP2 alters grey matter concentrations in schizophrenia patients. Neurosci Lett.

[35] Bacon C, Rappold GA (2012). The distinct and overlapping phenotypic spectra of FOXP1 and FOXP2 in cognitive disorders. Hum Genet.

[36] Shu W, Yang H, Zhang L, Lu MM, Morrisey EE (2001). Characterization of a new subfamily of winged-helix/forkhead (Fox) genes that are expressed in the lung and act as transcriptional repressors. J Biol Chem.

[37] Bruce HA, Margolis RL (2002). FOXP2: novel exons, splice variants, and CAG repeat length stability. Human Genet.

[38] Lai CS, Gerrelli D, Monaco AP, Fisher SE, Copp AJ (2003). FOXP2 expression during brain development coincides with adult sites of pathology in a severe speech and language disorder. Brain.

[39] Spiteri E, Konopka G, Coppola G, Bomar J, Oldham M, Ou J, Vernes SC, Fisher SE, Ren B, Geschwind DH (2007). Identification of the transcriptional targets of FOXP2, a gene linked to speech and language, in developing human brain. Am J Hum Genet.

[40] Vernes SC, Spiteri E, Nicod J, Groszer M, Taylor JM, Davies KE, Geschwind DH, Fisher SE (2007). High-throughput analysis of promoter occupancy reveals direct neural targets of FOXP2, a gene mutated in speech and language disorders. Am J Hum Genet.

[41] Vernes SC, Oliver PL, Spiteri E, Lockstone HE, Puliyadi R, Taylor JM, Ho J, Mombereau C, Brewer A, Lowy E (2011). Foxp2 regulates gene networks implicated in neurite outgrowth in the developing brain. PLoS Genet.

[42] Clovis YM, Enard W, Marinaro F, Huttner WB, De Pietri TD (2012). Convergent repression of Foxp2 3'UTR by miR-9 and miR-132 in embryonic mouse neocortex: implications for radial migration of neurons. Development.

[43] Tsui D, Vessey JP, Tomita H, Kaplan DR, Miller FD (2013). FoxP2 regulates neurogenesis during embryonic cortical development. J Neurosci.

[44] Poliak S, Peles E (2003). The local differentiation of myelinated axons at nodes of Ranvier. Nat Rev Neurosci.

[45] Strauss KA, Puffenberger EG, Huentelman MJ, Gottlieb S, Dobrin SE, Parod JM, Stephan DA, Morton DH (2006). Recessive symptomatic focal epilepsy and mutant contactin-associated protein-like 2. N Engl J Med.

[46] Abrahams BS, Tentler D, Perederiy JV, Oldham MC, Coppola G, Geschwind DH (2007). Genome-wide analyses of human perisylvian cerebral cortical patterning. Proc Natl Acad Sci U S A.

[47] Verkerk AJ, Mathews CA, Joosse M, Eussen BH, Heutink P, Oostra BA (2003). CNTNAP2 is disrupted in a family with Gilles de la Tourette syndrome and obsessive compulsive disorder. Genomics.

[48] Alarcon M, Abrahams BS, Stone JL, Duvall JA, Perederiy JV, Bomar JM, Sebat J, Wigler M, Martin CL, Ledbetter DH (2008). Linkage, association, and gene-expression analyses identify CNTNAP2 as an autism-susceptibility gene. Am J Hum Genet.

[49] Whitehouse AJ, Bishop DV, Ang QW, Pennell CE, Fisher SE (2011). CNTNAP2 variants affect early language development in the general population. Genes Brain Behav.

[50] O'Roak BJ, Deriziotis P, Lee C, Vives L, Schwartz JJ, Girirajan S, Karakoc E, Mackenzie AP, Ng SB, Baker C (2011). Exome sequencing in sporadic autism spectrum disorders identifies severe de novo mutations. Nat Genet.

[51] Li S, Weidenfeld J, Morrisey EE (2004). Transcriptional and DNA binding activity of the Foxp1/2/4 family is modulated by heterotypic and homotypic protein interactions. Mol Cell Biol.

[52] Ferland RJ, Cherry TJ, Preware PO, Morrisey EE, Walsh CA (2003). Characterization of Foxp2 and Foxp1 mRNA and protein in the developing and mature brain. J Comp Neurol.

[53] Teramitsu I, Kudo LC, London SE, Geschwind DH, White SA (2004). Parallel FoxP1 and FoxP2 expression in songbird and human brain predicts functional interaction. J Neurosci.

[54] Pariani MJ, Spencer A, Graham JM, Rimoin DL (2009). A 785 kb deletion of 3p14.1p13, including the FOXP1 gene, associated with speech delay, contractures, hypertonia and blepharophimosis. Eur J Med Genet.

[55] Hamdan FF, Daoud H, Rochefort D, Piton A, Gauthier J, Langlois M, Foomani G, Dobrzeniecka S, Krebs MO, Joober R (2010). De novo mutations in FOXP1 in cases with intellectual disability, autism, and language impairment. Am J Hum Genet.

[56] Zhang J, Webb DM, Podlaha O (2002). Accelerated protein evolution and origins of human-specific features: Foxp2 as an example. Genetics.

[57] Enard W, Przeworski M, Fisher SE, Lai CS, Wiebe V, Kitano T, Monaco AP, Paabo S (2002). Molecular evolution of FOXP2, a gene involved in speech and language. Nature.

[58] Li G, Wang J, Rossiter SJ, Jones G, Zhang S (2007). Accelerated FoxP2 evolution in echolocating bats. PloS One.

[59] Webb DM, Zhang J (2005). FoxP2 in song-learning birds and vocal-learning mammals. J Hered.

[60] Krause J, Lalueza-Fox C, Orlando L, Enard W, Green RE, Burbano HA, Hublin JJ, Hanni C, Fortea J, de la Rasilla M (2007). The derived FOXP2 variant of modern humans was shared with Neandertals. Curr Biol.

[61] Coop G, Bullaughey K, Luca F, Przeworski M (2008). The timing of selection at the human FOXP2 gene. Mol Biol Evol.

[62] Ptak SE, Enard W, Wiebe V, Hellmann I, Krause J, Lachmann M, Paabo S (2009). Linkage disequilibrium extends across putative selected sites in FOXP2. Mol Biol Evol.

[63] Maricic T, Gunther V, Georgiev O, Gehre S, Curlin M, Schreiweis C, Naumann R, Burbano HA, Meyer M, Lalueza-Fox C (2013). A recent evolutionary change affects a regulatory element in the human FOXP2 gene. Mol Biol Evol.

[64] Konopka G, Bomar JM, Winden K, Coppola G, Jonsson ZO, Gao F, Peng S, Preuss TM, Wohlschlegel JA, Geschwind DH (2009). Human-specific transcriptional regulation of CNS development genes by FOXP2. Nature.

[65] Haesler S, Wada K, Nshdejan A, Morrisey EE, Lints T, Jarvis ED, Scharff C (2004). FoxP2 expression in avian vocal learners and non-learners. J Neurosci.

[66] Bonkowsky JL, Chien CB (2005). Molecular cloning and developmental expression of foxP2 in zebrafish. Dev Dyn.

[67] Takahashi K, Liu FC, Hirokawa K, Takahashi H (2003). Expression of Foxp2, a gene involved in speech and language, in the developing and adult striatum. J Neurosci Res.

[68] Takahashi K, Liu FC, Oishi T, Mori T, Higo N, Hayashi M, Hirokawa K, Takahashi H (2008). Expression of FOXP2 in the developing monkey forebrain: comparison with the expression of the genes FOXP1, PBX3, and MEIS2. J Comp Neurol.

[69] Campbell P, Reep RL, Stoll ML, Ophir AG, Phelps SM (2009). Conservation and diversity of Foxp2 expression in muroid rodents: functional implications. J Comp Neurol.

[70] Shu W, Cho JY, Jiang Y, Zhang M, Weisz D, Elder GA, Schmeidler J, De Gasperi R, Sosa MA, Rabidou D (2005). Altered ultrasonic vocalization in mice with a disruption in the Foxp2 gene. Proc Natl Acad Sci U S A.

[71] Fujita E, Tanabe Y, Shiota A, Ueda M, Suwa K, Momoi MY, Momoi T (2008). Ultrasonic vocalization impairment of Foxp2 (R552H) knockin mice related to speech-language disorder and abnormality of Purkinje cells. Proc Natl Acad Sci U S A.

[72] Groszer M, Keays DA, Deacon RM, de Bono JP, Prasad-Mulcare S, Gaub S, Baum MG, French CA, Nicod J, Coventry JA (2008). Impaired synaptic plasticity and motor learning in mice with a point mutation implicated in human speech deficits. Curr Biol.

[73] Gaub S, Groszer M, Fisher SE, Ehret G (2010). The structure of innate vocalizations in Foxp2-deficient mouse pups. Genes Brain Behav.

[74] Kurt S, Fisher SE, Ehret G (2012). Foxp2 mutations impair auditory-motor association learning. PloS One.

[75] French CA, Jin X, Campbell TG, Gerfen E, Groszer M, Fisher SE, Costa RM (2012). An aetiological Foxp2 mutation causes aberrant striatal activity and alters plasticity during skill learning. Mol Psychiatry.

[76] Haesler S, Rochefort C, Georgi B, Licznerski P, Osten P, Scharff C (2007). Incomplete and inaccurate vocal imitation after knockdown of FoxP2 in songbird basal ganglia nucleus Area X. PLoS Biol.

[77] Teramitsu I, White SA (2006). FoxP2 regulation during undirected singing in adult songbirds. J Neurosci.

[78] Miller JE, Spiteri E, Condro MC, Dosumu-Johnson RT, Geschwind DH, White SA (2008). Birdsong decreases protein levels of FoxP2, a molecule required for human speech. J Neurophysiol.

[79] Teramitsu I, Poopatanapong A, Torrisi S, White SA (2010). Striatal FoxP2 is actively regulated during songbird sensorimotor learning. PloS One.

[80] Enard W, Gehre S, Hammerschmidt K, Holter SM, Blass T, Somel M, Bruckner MK, Schreiweis C, Winter C, Sohr R (2009). A humanized version of Foxp2 affects cortico-basal ganglia circuits in mice. Cell.

[81] Reimers-Kipping S, Hevers W, Paabo S, Enard W (2011). Humanized Foxp2 specifically affects cortico-basal ganglia circuits. Neuroscience.

